# Why and How Did Narrative Fictions Evolve? Fictions as Entertainment Technologies

**DOI:** 10.3389/fpsyg.2022.786770

**Published:** 2022-03-01

**Authors:** Edgar Dubourg, Nicolas Baumard

**Affiliations:** Département d’Études Cognitives, Institut Jean Nicod, ENS, EHESS, CNRS, PSL University, Paris, France

**Keywords:** cultural evolution, evolutionary psychology, fiction (narrative), fictionality, cultural attraction, superstimuli

## Abstract

Narrative fictions have surely become the single most widespread source of entertainment in the world. In their free time, humans read novels and comics, watch movies and TV series, and play video games: they consume stories that they know to be false. Such behaviors are expanding at lightning speed in modern societies. Yet, the question of the origin of fictions has been an evolutionary puzzle for decades: Are fictions biological adaptations, or the by-products of cognitive mechanisms that evolved for another purpose? The absence of any consensus in cognitive science has made it difficult to explain how narrative fictions evolve culturally. We argue that current conflicting hypotheses are partly wrong, and partly right: narrative fictions are *by-products* of the human mind, because they obviously co-opt some pre-existing cognitive preferences and mechanisms, such as our interest for social information, and our abilities to do mindreading and to imagine counterfactuals. But humans reap some fitness benefits from producing and consuming such appealing cultural items, making fictions *adaptive*. To reconcile these two views, we put forward the hypothesis that narrative fictions are best seen as *entertainment technologies* that is, as items crafted by some people for the proximate goal to grab the attention of other people, and with the ultimate goal to fulfill other evolutionary-relevant functions that become easier once other people’s attention is caught. This hypothesis explains why fictions are filled with exaggerated and entertaining stimuli, why they fit so well the changing preferences of the audience they target, and why producers constantly make their fictions more attractive as time goes by, in a cumulative manner.

## Introduction

Narrative fictions are the hallmark of modern culture. People all around the world spend an enormous and growing amounts of time consuming them, in the forms of novels, films, TV series, video games, manga, or theatre plays. For instance, humans in 22 different countries spend on average more time watching TV than doing sport, shopping, attending events or even seeing friends (Our World in Data, 2020). The production of narrative fictions has risen too, exponentially, both in number and in revenue, to tremendous levels. According to the latest estimations, the film industry and the book industry are worth more than 100 billion dollars worldwide each (Motion Picture Association, 2020), and the video game industry is worth 200 billons dollars alone (Accenture, 2021). The recent massive success of streaming platforms for films and TV series, such as Netflix and Disney+, is yet another cue of this far-reaching cultural phenomenon.

Yet, the questions of the origin and evolution of narrative fictions have constituted a puzzle for decades. Are such behaviors of producing and consuming narrative fictions biological adaptations, or by-products? How do narrative fictions culturally evolve? There is little consensus, nor any evidence of a search of consensus, as to how and why narrative fictions emerged in human cultures. In evolutionary sciences, the question is framed as followed: “How can it make evolutionary sense that members of a species successful enough to reshape the earth spend so much time in telling one another stories that neither tellers nor listeners believe?” (Boyd, 2018). Why fiction, then? Why did narrative fictions appear? Why are they appealing? And why are they more successful in modern societies? We first review a set of current hypotheses before proposing the “entertainment hypothesis,” which posits that fictions are best seen as entertainment technologies.

## State of the Current Hypotheses

### The Adaptive Hypotheses (and the Problem of Specificity)

A common view in behavioral approaches to literature is that the capacity to tell stories is adaptive (Gottschall and Wilson, 2005; Carroll, 2012). Notably, it has been argued that consuming fictions leads to acquire fitness-related knowledge (Sugiyama, 2001; Smith et al., 2017; Schniter et al., 2018; Nakawake and Sato, 2019; Sugiyama, 2021b), self-regulate one’s emotional states (Schaeffer, 1999; Gottschall and Wilson, 2005; Martin et al., 2018), simulate fake scenarios to be better prepared to face the real world (Tooby and Cosmides, 2001; Sugiyama, 2005; Mar and Oatley, 2008; Bloom, 2010; Gottschall, 2012; Clasen, 2019; van Mulukom and Clasen, 2021), or attract sexual mates (Miller, 2001). Evolutionary speaking, these hypotheses would hold only if our ancestors had faced a specific adaptive challenge that the behavior of producing or consuming fictions would have *specifically* solved (Tooby and Cosmides, 1992). It does not appear to be the case: neither of these hypotheses identify an adaptive function that is specific to narrative fictions (Carroll, 2012).

Let us take first the evolutionary theories which have proposed that the function of fictional narratives is to transmit adaptive information, be it about foraging (Sugiyama, 2001, 2021a), animals (Nakawake and Sato, 2019) or cooperation (Coe et al., 2006; Smith et al., 2017). The point is that such claims are not specific to narrative fictions, as factual narratives can also (and is, we assume, even more efficient to and more used to) transmit such generalizable knowledge. That is, communication and social learning had solved the adaptive problem of information transmission in such a way that fiction does not appear to enhance (Dunbar, 2003; Boyd et al., 2011; Boyd, 2018). Importantly, if many factual pieces of information, or ‘teaching moments’, as Sugiyama (2021b) call them, are embedded in fictions, they are precisely features that would make us doubt of the fictional status of the overall product. To put it in another way, if individuals truly wanted to convey relevant and important information about the world, they would not use invented content or pragmatic signals of fictionality (letting the consumers understand that the text is partly composed of falsehoods, for example, by introducing the story with “Once upon a time”). In a nutshell, we contend that telling an openly non-fictional story is a much more efficient way to transmit non-fictional information.

Similarly, the simulation hypothesis cogently explains why humans have the capacity to imagine or simulate hypothetical scenarios: because it solves the adaptive challenge of forecasting problems and working out solutions without actual practice (Oatley, 1999; Harris, 2000; Tooby and Cosmides, 2001). Imagination and foresight might be evolutionary adaptations (Suddendorf and Corballis, 2007; Fuentes, 2020; van Mulukom, 2020), but imagination is not fiction. Military strategists, projects managers, or engineers all need to imagine several potential futures in order to find the best options. But this activity does not fall under the category of fiction. In fact, a science fiction writer is likely to trade the credibility of a simulation for its potential in terms of entertainment, through the invention and ostensive exaggeration of attention-getting situations. In science-fiction, nuclear wars, aggressive IA, and alien invasion are the rigueur, *not* in military’s strategies related to actual potential threats. In other words, if speculative imagination aims at forecasting potential events to be better prepared to real life, this capacity seems now (and since quite a long time) to be used to invent and share narrative fictions that do not directly aim at being better prepared to face potential real situations, because they are too far from real events (Morin et al., 2019). From *A Brave New world* to *1984* to *The Neuromancer*, the worlds of science fiction are most often very dark, not because they aim at forecasting the future, but because they aim at entertaining the readers.

Other approaches posit that fictions are adaptive because they would train or enhance our social skills (Zunshine, 2006; Mar and Oatley, 2008). However, human social cognitive capacities, such as Theory of Mind (Gerrans, 2002; Tsoukalas, 2018), and human behavioral preferences, such as morality (Sperber and Baumard, 2012; Baumard et al., 2013; Tomasello, 2015), have been selected by natural selection and do not need fiction consumption to fully develop. If it was adaptive for individuals to be more cooperative, more cooperative individuals, that is individuals genetically more motivated to cooperate, would be naturally selected without the need to go through the implementation of the cognitive capacities needed for the behaviors of producing fictions.

To take a similar case, no one argues that humans need the consumption of fiction to enhance their sexual and romantic interests or to motivate themselves to raise children, because it is clear that if there were an adaptive advantage to being more motivated to have sex or to care for one’s children, then individuals who are more motivated to have sex or to care for their children would be naturally selected (Cosmides and Tooby, 2013). As a matter of fact, it is interesting to note that pornography or romance are seldom view as adaptive, despite the fact that they share the same fictional nature as other more legitimate fictions (Salmon, 2012). Besides, in the empirical literature, the effects of narrative fictions on the consumers’ beliefs or behavior are overall small (Gentile et al., 2009; Mulligan and Habel, 2013; Vezzali et al., 2015; Borum Chattoo and Feldman, 2017; Mumper and Gerrig, 2017; Dodell-Feder and Tamir, 2018; Rathje et al., 2021). Importantly, they are also elicited by *factual* narratives (Barnes and Black, 2021). For instance, studies comparing people who consumed fictional movies and others who watched documentaries found no evidence of differential effects on prosociality (LaMarre and Landreville, 2009).

The same counterarguments hold for the hypothesis that narrative fictions have the adaptive function to regulate one’s emotional states. Emotional instability derives from hard-wired cognitive mechanisms that make people react to various situations in adaptive ways (Cosmides and Tooby, 2000; Nettle, 2012; Al-Shawaf et al., 2016). More precisely, emotions, such as fear, shame, guilt, gratitude, or pride, are cognitive programs whose specific function is to coordinate other mechanisms that should be efficiently coordinated facing a specific (adaptive) problem (Al-Shawaf et al., 2016). These behavioral programs are already fine-tuned to each situation, so that fictions would in fact be counterproductive in terms of biological fitness if they substantially impacted their regulation. However, we do not make the (absurd) claim that narrative fictions do not change the emotions of the consumers. We rather argue that they did not *evolve to* perform such a function. And, again, this idea is supported by the fact that the (minor) affective effect is not fiction-specific: it is elicited, for instance, by music (Mehr et al., 2020; Savage et al., 2020).

To conclude this section, we argue that the current adaptive hypotheses do not account for the fact that producers *invent* narratives and do not explain why consumers pay attention to narrative. The proposed evolutionary functions are not specific to fictional narratives. It is not clear why humans would need to evolve fictions to regulate their emotions, transmit information, or forecast the future because evolving fiction would not be the most straightforward way to do that. Also, adaptive hypotheses do not explain why such fictions should depart from realistic narratives. Because fictional narratives do exist in human cultures, there should be a *specific* advantage for narratives to be *fictional*.

### The By-product Hypothesis (and the Problem of Fitness Benefits)

The other hypothesis posits that narrative fictions are by-products, and therefore did not evolve through natural selection. Within this framework, it is argued that fictions co-opt pre-existing cognitive capacities and preferences that evolved in the human mind for no reasons related to fictions, and that this explanation is sufficient to explain the existence, universality, and pervasiveness of fictions in human cultures. A version of this hypothesis has been famously called the “cheesecake hypothesis” by Pinker (1997). Cheesecakes exploit the cognitive mechanisms designed to make humans detect and like the taste of glucose, at the proximate level. Those mechanisms have been selected by evolution because the ingestion of glucose enhanced fitness in the environments in which the human mind evolved, at the ultimate level (Ramirez, 1990). Therefore, the preference that makes humans like cheesecakes evolved long before cheesecakes appeared. Masks are another good example: because they display visual patterns that are close to real faces (e.g., two points at the top of a round shape, one point at the bottom of the same round shape; Farroni et al., 2005), they meet the input conditions of the face recognition mechanism that evolved to identify individuals and understand their emotions (Sperber and Hirschfeld, 2004). As a consequence, they artificially trigger people’s face recognition mechanism and automatically grab their attention. On top of that, by exaggerating facial traits and facial expressions (e.g., bigger eyes, more colorful faces, etc.), they produce new, original, and often more powerful emotions.

Likewise, fictions would be pleasurable and attention-grabbing for the human mind, at the proximate level, because they would co-opt a myriad of cognitive preferences, that evolved before symbolic culture even emerged. In line with the by-product hypothesis, many studies brought evidence that fictions do co-opt cognitive preferences that evolved before fictions even existed (Table 1).

**Table 1:** Research papers explaining the appeal of fictions by linking fictional traits with the cognitive mechanisms they co-opt, and the evolutionary function of the mechanisms.

Research paper	Fictional feature	Cognitive preference	Adaptive function
**The psychological foundations of the hero-ogre story (Jobling, 2001)**	Opposition between a hero and a monster committing crimes against the ingroup	Negative bias in the perception of outgroup members	Removing empathy toward potential enemies
**Explaining the origins of comedy and tragedy (Nettle, 2005a)**	Social networks with status competition and mate selection	Mechanisms designed to observe and track interpersonal behaviors	Making behavioral decisions conducive to high status or mate choice
**A Biological Homage to Mickey Mouse (Gould, 2008)**	Young protagonists with big heads (relative to their bodies) and dotting eyes	Mechanisms designed to detect and pay attention to baby faces	Ensuring parental care and investment
**High on Crime Fiction and Detection (Grodal, 2010)**	Crime fictions with a focus on the rational path to the truth, and the protagonists investigating	Cue-based seeking system	Foraging and hunting
**The rape-revenge film: biocultural implications (Andrews, 2012)**	A rape (or another violent act) motivates an act of vengeance	Preference for retributive justice	Keeping potential offenders in check
**Monsters Evolve: A Biocultural Approach (Clasen, 2012)**	Horrific monsters in horror fictions	Mechanisms designed to detect and evaluate predators	Avoiding predators, fleeing
**Evil Origins: A Darwinian Genealogy of the Popcultural Villain (Kjeldgaard-Christiansen, 2016)**	Archetypal anti-social, selfish, dominant and/or sadistic villains	Free-rider detector system	Avoiding free-riders and cheaters in the biological market of cooperation
**The evolutionary and psychological foundations of universal narrative structure (Singh, 2019)**	Protagonists depicted as cooperative partners which are competent, warm and/or in need for help	Mechanisms designed to assess others’ power and will to reciprocate	Ensuring cooperation by partner choice
**Why Imaginary Worlds? The cultural evolution of imaginary worlds in fictions (Dubourg and Baumard, 2021)**	Imaginary worlds with invented spatial environments	Exploratory preferences and abilities	Motivating spatial exploration and the discovery of fitness-enhancing resources

The by-product hypothesis explain well why fictions are attention grabbing (they meet the input conditions of many preexisting cognitive mechanisms). However, it does not explain why (1) producers produce fictions (what is the fitness advantage of creating worlds, characters, and plots?) and (2) why consumers consume fictions (what is the fitness advantage of spending so much time to learn about worlds, characters, and plots that do not exist?; André et al., 2020).

To put it in other words, the by-product hypothesis makes a strong hypothesis about the nature of human cognition. It indeed assumes that the interest in fictions is essentially a mismatch and that humans are not able to understand that they are wasting their time with imaginary characters and imaginary worlds. This is very possible in theory. For instance, pure psychoactive drugs (e.g., heroin) that are administered directly in the blood are very novel. They thus bypass adaptive information-processing systems and induce positive emotions that give a false signal of a fitness benefit. This signal hijacks mechanisms of “liking” and “wanting,” and is inherently pathogenic (Nesse and Berridge, 1997). However, even in the case of drug, this assumption should be considered with caution. For instance, the consumption of drugs that are eaten or smoked may very well be adaptive, notably against parasites. In line with the adaptive framework, the consumption of these drugs is lower for individuals whose brain is not mature enough to tolerate neurotoxins, and therefore adaptively varies with age, sex, and condition (e.g., pregnancy; Hagen et al., 2013). To conclude, it is possible that fictions are just the result of a mismatch, and hijack evolutionary ancient mechanisms. However, this mismatch hypothesis should really be used in the last resort (as in the case of directly administered drugs) when all possible explanations have failed. We believe that this is not the case for fictions.

In the next section, we propose a middle-ground solution that explains why the existence of fiction is adaptively plausible, for both the producers and the consumers, and why the content of fiction is so well tuned to the human mind.

## A Middle-Ground Solution: Fictions as Culturally Evolved Technologies to Fulfill Adaptive Goals

We hypothesize that fictions are best seen as technologies. Why technologies? Fictions have a lot in common with other cultural inventions such as kayaks, wheels, or computers, which all are human technologies: they are cultural products designed by the human mind to perform specific functions (Stout, 2021). Have humans evolved cognitive mechanisms *specifically* designed to craft kayaks? The obvious answer is no: we rather evolved (1) specific motivations (e.g., to get food and to get status) that regulate how we allocate our time and energy (Cosmides and Tooby, 2013) and (2) specific cognitive mechanisms (e.g., planification, hand-eye coordination, and fine motor skills) that are flexible enough to be used in a variety of contexts (Vaesen, 2012; Osiurak and Reynaud, 2019). This led to crafting kayaks, for instance, because they meet the purpose of travelling on water, in order to fulfill evolved motivations such as getting food, meeting social partners, and exploring new places.

This reasoning also applies to symbolic culture such as alphabets (Dehaene, 2004; Changizi et al., 2006; Morin et al., 2019), fake-news (Altay et al., 2020), shamanism (Singh, 2018), make-up (Sperber and Hirschfeld, 2004), puritanical norms (Fitouchi et al., 2021), and symphonic orchestra (Mehr et al., 2020; Dubourg et al., 2021c). For instance, painters have discovered that, for some population, direct gaze (Morin, 2013) and “neotenic” features (big eyes or round faces; Costa and Corazza, 2006) in portraits are likely to attract the viewer’s attention, which is we argue the ultimate motivation of painters. What should be considered as adaptive, then, is the *use* of kayaks, computers, portraits, and other cultural productions to fulfill fitness enhancing goals (André et al., 2020; Singh, 2020). Following the same line of argument, we argue that humans did not evolve any specific mechanisms to invent fictions, but rather used their evolved cognitive mechanism to invent fictions just as they did for any other technologies. Yet, the production of fictions can be considered as an adaptive behavior because it is regulated by the evolved motivation to fulfill a specific adaptive goal. What is this goal? We argue that fictions are specifically used to entertain other people.

## A Specific Kind of Technologies: Entertainment Technologies

### The Centrality of Entertainment in Fictions

Literary theorists and historians have long noticed the cross-culturally recurrent and entertaining features of fictions (which have also been called “themes,” “tropes,” or “patterns”) such as adventures, conflicts, love stories, imaginary worlds, monsters, gossip, authority, success, and the search of social status (Kato and Saunders, 1985, p. 232; Pavel, 1986, pp. 147–148; Campbell, 1993; Schaeffer, 1999, p. 241; Huang, 2001, pp. 60–61; Hogan, 2003; Booker, 2004). Evolutionary critics in the humanities and evolutionary social scientists brought evidence that such universal fictional features are influenced by the evolutionary history of the human mind (Carroll, 1995; Gottschall, 2008; Fisher and Salmon, 2012; Saad, 2012; Grodal, 2017). More recently, as we have seen in section The By-product Hypothesis (and the Problem of Fitness Benefits), these cross-cultural features have been linked to specific cognitive preferences (Table 1). In all, there seems to be a large and interdisciplinary consensus to say that narrative fictions include attractive and entertaining features. The question therefore is: Why are such features attractive and entertaining to the human mind?

We contend that such pleasurable features of fictions are very close to what evolutionary biologists called *superstimuli* (Tinbergen, 1969; Barrett, 2010). Many studies show that some species, in the course of their evolutionary history, recycled pre-existing attractive traits for new evolutionary relevant functions such as attracting mates (Lorenz, 1966; Krebs and Dawkins, 1978; Basolo, 1990; Ryan et al., 1990). For instance, because the female frog *Physalaemus pustulosus* had developed preferences for lower-frequency chuck sounds, males evolved the ability to produce such sounds to tap into this sensory preference (Ryan et al., 1990).

In nonhuman animals, this recycling of preexisting preferences usually emerges through *biological* selection. In humans, it can emerge through cultural evolution: producers use their expertise to target and refine stimuli that are already appealing to consumers (Lightner et al., 2022), so as to fulfill fitness relevant goals (Singh, 2020). We will explain what these goals are in the next sub-section.

We therefore argue that content features in fictions are superstimuli: they are crafted to resemble stimuli that were *already* appealing to the human mind, because of the natural selection of attention-orienting cognitive mechanisms, and of the pleasure systems rewarding the behavior of paying attention to such stimuli. This is a form of what psychologists have called “content-based attraction,” when the attraction and prevalence of a cultural item is favored by its content (Sperber, 1996; Claidière and Sperber, 2007; Scott-Phillips et al., 2018).

A question follows: Why are such stimuli attention-grabbing in the first place (in the real world)? This is where we fall back on the by-product hypothesis: such preferences for some stimuli (e.g., social information) evolved because humans endowed with them survived and reproduced better in the ancestral environments when the human cognition evolved.

In evolutionary and cognitive approaches to fictional content, superstimuli have already been studied in fictional texts (Jobling, 2001; Nettle, 2005a,b; Singh, 2019), in movies (Cutting et al., 2011; Andrews, 2012; Clasen, 2012; Cutting, 2016, 2021; Sobchuk and Tinits, 2020), in video games (Jansz and Tanis, 2007; Mendenhall et al., 2010), in artistic representations (Verpooten and Nelissen, 2010, 2012), and in cross-media approaches to fiction (Grodal, 2010; Barrett, 2016; Dubourg and Baumard, 2021). Let us note that such fictional superstimuli can be narrative superstimuli (e.g., how Marcel in *Search of Lost Time* reaches prestige), visual superstimuli (e.g., the form of Mickey), auditory superstimuli (e.g., the terrifying sounds in horror films), and other sensory superstimuli (e.g., the sense of control in open-world video games or in virtual reality games). Producers of fictions use any means available to them to make the most attention-grabbing superstimuli and therefore the most entertaining fictions.

Of course, the pleasure-inducing effect elicited by superstimuli in fictions is also elicited by some other cultural behavior and products, such as sport and news (Barrett, 2010, 2016). This is because the fiction industry is not the only one to target entertainment. However, the presence of superstimuli successfully isolate fiction from non-fiction, because superstimuli are never included in non-fictional narratives: the obligation to (try to) stick to real facts prevent, to a large extent, producers of non-fictional narratives to invent and exaggerate any feature (or else their epistemic reputation might suffer, and the benefits of attracting other people’s attention would be overweighted by the reputational costs of having deceived their audience). We contend that such a distinction is intuitive to consumers: they will continue to consume and positively evaluate fictions that they take pleasure from, while they will either stop consuming or negatively evaluate fictions that deceive the expectation to be entertained. Conversely, when they consume non-fictional narratives, such as a philosophical treatise, a political essay, or an history documentary, their primary goal is to learn things, so that they will not stop consuming the non-fiction if they are not entertained, and they will not base their evaluation on this criterion.

### The Fitness Consequences of Entertainment Technologies

Why would producing fictions be adaptive? With the entertainment hypothesis, this question is the same as the following one: Why would attracting the attention of other people by inventing entertaining cultural items should bring any fitness benefit? We propose that, because they are highly attractive and entertaining, fictions can be used to fulfill any evolutionary relevant goal that needs others’ attention to be caught, be it signaling one’s values to potential mates (Miller, 2001) or cooperative partners (Bourdieu, 2010; André et al., 2020; André and Baumard, 2020; Dubourg et al., 2021b; Lightner et al., 2022), transmitting knowledge (Schniter et al., 2018; Nakawake and Sato, 2019; Sugiyama, 2021b), communicating social norms (Mar and Oatley, 2008; Ferrara et al., 2019), or selling products (Saad and Gill, 2000; Saad, 2012).

Consistently, narrative fictions seem to have been used (1) as recruitment technologies: they allow the producers of fictions to attract and potentially cooperate with individuals that matter to them, by signaling one’s qualities (e.g., their competence, their moral sense, and their intelligence) and therefore enhancing one’s reputation as a cooperative partner (Sperber and Baumard, 2012). For instance, in many countries at most time in history, cultural institutions and organizations aimed at spotlighting the producers of fictions, from the poetry contests (*uta-awase*) in Japan from the Heian period to the modern Nobel Prize in Literature and movie Academy Awards. Narrative fictions are also obviously used to (2) derive economic or material gains. This is clearly pictured in the form fiction production and fiction consumption took in large-scale societies, that of a massive (and highly lucrative) contract-based market.

Crucially, such adaptive goals need not be conscious or deliberate. They need not be the only motivations either: drawing on adaptive hypotheses that we reviewed in section State of the Current Hypotheses, producers of fictions can have other goals, such as transmitting knowledge (Sugiyama, 2021a). The association between both motivations of educating and entertaining people has produced a new form of cultural devices called “Edutainment” (Singhal, 2004; Anikina and Yakimenko, 2015), which we argue has emerged far back in human cultural history, embedding not only recent fictions (e.g., *Dora the Explorer*), but also ancient folktales (Sugiyama, 2021b) and other literary forms such as pre-17^th^ century European fairy tales.

According to this hypothesis, narrative fictions are sustained because they confer fitness benefits to the consumers too. First, let us note that the opportunity costs of fiction consumption seem rather low because people do not seem to consume fictions at the expense of other more “evolutionary relevant” activities such as sleeping, eating, and parenting. On the other hand, consumers can use fictions they liked to signal their skills (Veblen, 1899; Bourdieu, 1979; Lizardo, 2006, 2013). They can also use more culturally successful fictions they liked to signal their personality traits (Dubourg et al., 2021a), or to share cultural focal points for social coordination (Dubourg et al., 2021b,c). Besides, human minds have evolved specialized cognitive mechanisms to detect and use social markers for coordination (Nettle and Dunbar, 1997; Boyer, 2018). We propose that preferences for fictions have become relatively important markers in the ecology of modern cultural diversity, because of their signaling potential.

### Summary of the Hypothesis

In all, we propose that humans did not specifically evolve the capacity to tell fictional stories, but they rather produce fictions thanks to a range of other adaptations (e.g., language, the capacity to simulate, Theory of Mind, and communicative inferences; Zunshine, 2006; Mellmann, 2012; Wilson, 2018). Yet, we do not consider fictions as “by-products,” because they clearly confer fitness benefits to the producers (André et al., 2020). We argue that fictions are “entertainment technologies” (Dubourg and Baumard, 2021): they are crafted by storytellers to artificially attract the attention of other people and then fulfill evolutionary-relevant goals (Singh, 2020). Obviously, fictions are not the only example of entertainment technologies. Sport, TV shows (Barrett, 2010, 2016), music (Dubourg et al., 2021a), and performing arts (Verpooten and Nelissen, 2010, 2012) are also entertainment technologies in the sense that they are created to trigger people’s attention, and are consumed because they exaggerate the features of phenomena (e.g., human voice and interindividual competition) that humans evolved to be interested in.

## The Cultural Evolution of Fictions

The main question which remains is whether this account of the evolutionary origin of narrative fictions can explain *how* they culturally evolved. If such fictions emerged because producers aim at entertaining their consumers by picking the locks of their cognitive preferences, we should observe that: (1) the cultural evolution of fictions is driven by the evolution of the consumers’ preferences (i.e., what best attracts their attention in specific conditions) and (2) the producers improve their productions by making them more attention-grabbing and pleasurable, in a cumulative manner.

### The Variability of Biological Preferences

The entertainment hypothesis posits that people’s preferences are factors of attraction and thus drive the cultural evolution of fictions, because the producers of fiction’s goal is to make entertaining cultural products. Therefore, because people’s preferences vary, we expect narrative fictions to vary accordingly. More precisely, our framework predicts that the variability of preferences, which is explained and predicted by evolutionary psychologists and behavioral ecologists, impact the variability of cultural consumption. Here, we identify three main sources of the interdividual variability of evolved preferences: the life stage, the sex, and the conditions of the local ecology of the individuals. We propose that such causal factors of the variability of biological preferences can account for the cultural distribution of fictions across time and populations.

#### Life Stage

In humans, each life stage from infancy to old age (including childhood, juvenility, adolescence, and adulthood) has a specific suite of preferences, adaptively suited to the specific challenges they faced in the human evolutionary history (Bjorklund and Pellegrini, 2000; Del Giudice et al., 2009). As life-history theory puts it, natural selection has favored individuals who are able to adopt an optimal scheduling of preferences, so as to maximize their expected fitness (Hill, 1993; Kaplan and Gangestad, 2005; Gangestad and Kaplan, 2015). For instance, in every evolutionary model of human ontogeny, the life stages of childhood and juvenility are defined as learning periods for foraging skills (Kaplan et al., 2000; Kaplan and Robson, 2002) or social skills (Flinn and Ward, 2005) which is made possible by parental caregiving investments (compensating for the low productivity of younger individuals). This gives children the crucial opportunity to be explorative and curious (Gopnik et al., 2015; Gopnik, 2020), and crucially more so than adults (Defeyter and German, 2003; Gopnik et al., 2017; Blanco and Sloutsky, 2019; Sumner et al., 2019; Liquin and Lombrozo, 2020; Spreng and Turner, 2021). On the other hand, children and juveniles are still sexually immature. Juvenility is seen as a developmental (hormonal and psychological) switch leading to adolescence: behavior and preferences (adaptively) start to be shaped by sexual selection from this point onward (Del Giudice et al., 2009). For instance, in 11 different countries from around the world, risk-seeking preferences follow the same inverted-U pattern, peaking at around age 19 (Steinberg et al., 2018). Why? It is part of a broader reproductive strategy suited for the life stage of adolescence when humans become sexually mature and ought to signal their strength and resilience to costs to potential mates and rivals (Del Giudice et al., 2009).

Both examples we arbitrarily chose [that, overall, (1) children and juveniles have stronger exploratory preferences, and that (2) adolescents have stronger risk-oriented preferences] are only two examples among many other adaptive age-specific preferences. Both of them lead to predictions about age-specific cultural preferences. The basic idea here is that there exists such a thing as a *life history of cultural preferences*. For instance, in a previous work, we argued that imaginary worlds in fictions tap into our exploratory preferences, and we therefore predicted that such imaginary worlds (e.g., Tolkien’s Middle-Earth and Rowling’s Wizarding World) should be preferred by younger individuals (Dubourg and Baumard, 2021). Likewise, following our second example, we (more straightforwardly) predict that adolescents will prefer fictions with romantic and sexual stories as well as fictions with risk-seeking protagonists. Such predictions (among many others relying on the same line of argument) remain to be thoroughly tested with computational or experimental methods, so as to explain a part of the variability of cultural preferences for different fictions with insights from evolutionary developmental psychology.

#### Sex

Each sex faced specific adaptive problems and natural selection has favored different preferences to take them up (Trivers, 1972; Symons, 1981; Buss, 1995). This is particularly the case in the domain of mating strategy (Buss, 1994) and parenting investment (Bjorklund and Jordan, 2013; Wilcox and Kline, 2013). For instance, because human females invest more in their offspring than males (the minimum parental investment for a woman is 9-months pregnancy and several years of breastfeed), sexual selection resulted in females being more discriminating and males being more competitive (Trivers, 1972; Saad and Gill, 2001; Stewart-Williams and Thomas, 2013). Besides, specific courtship displays have evolved in both sexes as a result of mate preferences in one sex or the other, and this led to specific systems to detect such ornament-like features (Miller, 2001). For instance, females tend to seek more long-term commitment and a propensity to bring in resources (to ensure paternal caregiving investment).

Therefore, we argue that sex is another biological source of interdividual differences in cultural preferences. For example, following the evolutionary insight according to which female humans have sex-specific evolved mating preferences, it might be possible to predict which kind of romance fictions women will prefer. Cox and Fisher (2009) predicted that the success of popular romance novels from the widely successful Harlequin’s collection should be shaped by the evolved mating interests of women (accounting for 90% of the readers of Harlequin novels). They analyzed the titles of more than 15,000 novels from that collection and found that the 20 most frequent words in such titles were related to long-term romantic commitment. Sex-specific evolved mating preferences are also the cornerstones of classical romance novels such as Austen’s *Pride and Prejudice* (Strout et al., 2010), of highly popular ‘slash’ fictions (Salmon and Symons, 2004), and of traditional folktales from around the world (Gottschall et al., 2004).

Of course, there is also variability in how much such sex-related differences in preferences are pronounced. For instance, in economically developed countries, males tend to invest more resources in their offspring and to be more involved in long-term committed relationship (Geary, 2000). This evolution predicts that, since their life history get closer to the life history of females (e.g., high parental investment and preference for long-term relationships), their associated preferences should get closer to that of females (Stewart-Williams and Thomas, 2013). This observation leads to the prediction that men and women should like the same types of family-related fictions in ecologies in which sex-related differences (adaptively) fade away. Many more predictions can be derived and tested about the impact of sex-specific preferences on the cultural preferences for narrative fictions.

#### Local Ecology

**Figure 1:**
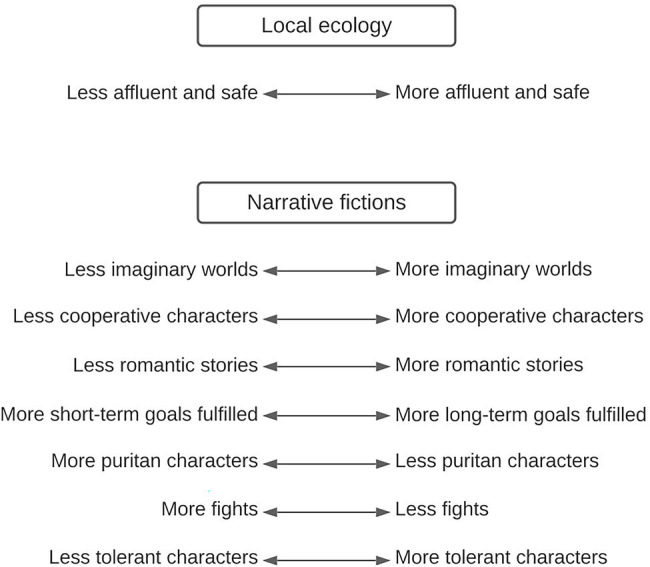
Examples of predictions about fictional features depending on the environment, derived from human’s adaptive phenotypic plasticity.

Finally, behavioral sciences have shown that some cognitive preferences adaptively vary in response to changes in the local environment, especially changes in the level of resources (Frankenhuis et al., 2016; Pepper and Nettle, 2017; Baumard, 2019; de Courson and Baumard, 2019; Mell et al., 2019; Boon-Falleur et al., 2020; De Courson and Nettle, 2021). For instance, higher levels of affluence, predictability and safeness makes people more future-oriented (Mell et al., 2019; Boon-Falleur et al., 2020; Guillou et al., 2020), more optimist (Nettle, 2012; Inglehart, 2020), more cooperative (Baumard, 2019; Jacquet et al., 2019), more tolerant (Inglehart, 2018), more romantic (Baumard et al., 2021; Martins and Baumard, 2021), and more explorative (Eliassen et al., 2007; Maspons et al., 2019; Gopnik, 2020). Improvements of living standards in human history, and in a wide range of different cultures, have indeed re-shaped many preferences in directions that are very consistent with this evolutionary account. Let us note that this plasticity in individuals’ preferences is considered to be an adaptation to environmental variation in that it allows them to adaptively fit their preferences to each specific ecology. It is called *adaptive phenotypic plasticity* (Figure 1).

Although it has been overlooked in evolutionary and psychological approaches to symbolic culture and cultural artefacts, we argue that this source of variability can explain a significant part of the variability of cultural preferences for fictions. Under the same rationale as we used for other sources of variability (that producers make fictions that please their audience at a given time, in a given location), we propose that adaptive phenotypic plasticity is a major causal explanation for the cultural evolution of fictions across time (in diachrony) and for the cultural distribution of fictions across countries or regions of the world (in synchrony). For instance, in more affluent societies, across both time and space, humans produced fictions with more romantic love stories (Baumard et al., 2021; Martins and Baumard, 2021), more cooperative relationships (Martins and Baumard, 2020), and more imaginary worlds (Dubourg et al., 2021a). This is the case because such elements tap into preferences that are more evoked in affluent environments. There is an avenue for a theory-driven, data-rich research program on the cultural evolution of fictions.

For instance, why would love stories such as *Romeo and Juliet* and fictions with imaginary worlds such as *The Lord of the Rings* be more successful at some times in history, and not at other times, and why in some regions of the world, and not others? In other articles, we reviewed evidence that (1) romantic love stories are attractive because they exploit the evolved emotional device designed to facilitate pair-bonding and parental investments (Fletcher et al., 2015; Baumard et al., 2021) and (2) imaginary worlds tap into exploratory preferences which have evolved in humans to prompt them to explore their environments and find evolutionary-relevant resources, such as food and shelter (Cohen et al., 2007; Dubourg and Baumard, 2021). We argued that both love-related preferences and exploratory preferences vary according to ecological conditions: in more affluent environments, people can afford to invest more in their family (both in their romantic relationships and in their children) and to be more explorative. As a result, adaptive phenotypic plasticity adaptively promotes such preferences in such fine-tuned local ecologies (Baumard et al., 2021; Dubourg and Baumard, 2021). We therefore predicted and provided evidence for the fact that romantic love stories and imaginary worlds increase when living conditions improve with economic developments (Baumard et al., 2021; Dubourg et al., 2021d; Martins and Baumard, 2021).

### The Cumulative Cultural Evolution of Fictions

Not only are narrative fictions filled with appealing stimuli, but we also posit that, over time, such stimuli are selectively retained and cumulatively refined to better attract the attention of the consumers. The basic idea is that narrative fictions compete for the attention of consumers in what one could call an ‘entertainment economy’. Producers are therefore likely to intensify already appealing stimuli, to increase the success of their narrative fictions. Importantly, producers need not know the evolutionary origins of such and such preferences shaping the content of their creations. The selection and refinement of features at each generation and the ‘trial and error’ process are sufficient to explain the improvement of fictional features across time.

Some empirical findings suggest that this is the case. The most grounded example is undoubtedly the one of the ‘baby schema’ in visual fictions. The ethologist Konrad Lorenz hypothesized that a set of infantile features, such as a round face and big eyes, is perceived as ‘cute’, at the proximate level, because, at the ultimate level, having one’s attention caught by cute babies motivated parental caregiving investments and was therefore adaptive. Lorenz (1943) provided correlational evidence that this was the case. More recently, experimental research reached the same conclusion: pictures of babies that were parametrically manipulated to produce an enhanced baby schema (e.g., with rounder faces and larger eyes than real babies) were rated as cuter and as motivating more caregiving than photographs of babies that were both manipulated to produce low baby schema (e.g., with less round faces and smaller eyes) and photographs not manipulated at all (Glocker et al., 2009a). Using the same pictures (as experimental stimuli) and functional magnetic resonance imagining, another study showed that a specific brain system (the mesocorticolimbic system) is responsible for the emotional and behavioral response to cute babies (Glocker et al., 2009b). This line of research provide straightforward predictions, when applying the cumulative cultural evolution framework to the entertainment hypothesis: if producers of fictions select, refine and exaggerate appealing stimuli (to better tap into evolved preferences and make more entertaining fictions), protagonists should become cuter and cuter. This has been empirically shown with Walt Disney’s Mickey: the evolution of its design is driven by this preference for cute baby faces: across the last decades, Mickey progressively became cuter, that is, more baby-like, with larger heads and more doting eyes (Hinde and Barden, 1985; Gould, 2008).

Recent empirical work started to unveil other cumulative processes in the refinement of entertaining features in fictions. Godzilla grabs our attention because its height and strength would make it a very dangerous predator if it were real, and a quick look at its successive representations shows that it gets bigger and taller over time (Dominy, 2019; Sobchuk, 2019). More universally, movies grab our attention in part because the rapidity of the sequence of shots (i.e., the shot lengths) is suited to make the eye movements reevaluate each visual depiction: with 75 years of Hollywood film, Cutting et al. (2011) provided empirical evidence that shot lengths have significantly decreased, to enhance this control over the audience’s eye movements. In another work, we argued that imaginary worlds cumulatively evolved too, by including more and more information background and information devices that modern consumers find attractive (Dubourg and Baumard, 2021). The examples taken here all show cumulative processes: producers at each generation selectively retain and cumulatively refine fictional elements that seem to best fit their goal of entertaining their audiences. We suspect that many more superstimuli in fictions have been cumulatively refine in recent times, because of the tremendous growth of both fiction production and consumption allowing faster cumulative processes. Much more empirical research is needed to assess the way each superstimulus in narrative fictions is cumulatively selected and refined over cultural history.

## Conclusion

We hypothesized that narrative fictions are neither adaptations nor by-products: they are entertainment technologies, that is, crafted cultural items that producers create to attract the attention of the consumers, entertain them, and fulfill other evolutionary-relevant goals (e.g., reputational benefits and economic gains). In doing so, producers of fictions use superstimuli (i.e., already appealing stimuli which are exaggerated in the fictions so as to make them even more appealing). We summarized external evidence that this is the case, from literary historians stating that some features are universal because they entertain their audience, to evolutionary social scientists arguing that the evolved mind has shaped the content of stories, and finally to evolutionary psychologists, who started to associate specific fictional features to specific evolved cognitive preferences. Finally, we argued that this hypothesis cogently explains how narrative fictions culturally evolve. First, because producers compete for the attention of the consumers, they should try to target specific cognitive preferences, which are age-, sex-, and context-specific. That is, biological determinants shape preferences and thus drive the distribution of fictions across time and population. Then, for the same reasons, producers at each new generation want to improve their narrative fictions by making them more attention-grabbing: they selectively retain and cumulatively refine appealing fictional features, in a cumulative manner. Overall, this hypothesis explains why and how narrative fictions evolved.

## Data Availability Statement

The original contributions presented in the study are included in the article/supplementary material, further inquiries can be directed to the corresponding author.

## Author Contributions

ED and NB conceived the main idea, designed the outline, and worked on the final paper. ED wrote a first draft. All authors contributed to the article and approved the submitted version.

## Funding

This work was supported by the FrontCog funding (ANR-17-EURE-0017).

## Conflict of Interest

The authors declare that the research was conducted in the absence of any commercial or financial relationships that could be construed as a potential conflict of interest.

## Publisher’s Note

All claims expressed in this article are solely those of the authors and do not necessarily represent those of their affiliated organizations, or those of the publisher, the editors and the reviewers. Any product that may be evaluated in this article, or claim that may be made by its manufacturer, is not guaranteed or endorsed by the publisher.
